# Sustainable Solutions for Eye Drop Plastic Waste: Challenges, Innovations, and Environmental Impact

**DOI:** 10.1155/joph/7375211

**Published:** 2025-11-27

**Authors:** Siddharth Gandhi, Michael Balas, Amandeep Rai

**Affiliations:** ^1^Faculty of Medicine, Queen's University, Kingston, Ontario, Canada; ^2^Department of Ophthalmology and Vision Sciences, University of Toronto, Toronto, Ontario, Canada; ^3^Kensington Eye Institute, Toronto, Ontario, Canada

**Keywords:** eye drops, green ophthalmology, ophthalmology, plastic waste, recycling

## Abstract

The widespread use of single-use plastic eye drop bottles in ophthalmology presents a significant environmental challenge. While essential for preventing contamination and ensuring patient safety, these bottles contribute to plastic waste due to their small size, multimaterial composition, and inadequate recycling infrastructure. This review examines the environmental impact of eye drop packaging across various ophthalmic conditions, including dry eye disease, cataract surgery, and glaucoma, highlighting the substantial cumulative burden of plastic waste and carbon emissions. The manuscript explores innovative solutions to mitigate this environmental footprint without compromising clinical standards. Key strategies discussed include product redesign using biodegradable materials, the adoption of multidose preservative-free systems, and the development of alternative drug delivery technologies like punctal plugs. Additionally, the importance of advanced recycling initiatives, such as specialized take-back programs, is emphasized. The paper also underscores the need for behavioral, institutional, and policy interventions, including educational campaigns, sustainable procurement practices, and the implementation of Extended Producer Responsibility (EPR) policies. By integrating these multifaceted approaches, the field of ophthalmology can align the critical priorities of patient safety and environmental sustainability, fostering a transition towards greener practices while maintaining high standards of care.

## 1. Introduction

The widespread use of single-use plastics has significantly impacted healthcare, including ophthalmology [1, 2]. Disposable eye drop bottles play a critical role in preventing infections and managing various ocular conditions [3]. However, their extensive use has also contributed to a growing environmental burden, particularly through plastic waste generation. While maintaining infection control remains essential, the increasing global concerns surrounding plastic pollution necessitate a reassessment of current practices, especially in high-volume fields such as ophthalmology [1–7].

This review examines the challenges associated with single-use eye drop bottles in ophthalmology. It explores their historical development, regulatory considerations, and environmental impact while identifying key limitations in current waste management systems. Additionally, it evaluates potential solutions, including material innovations, recycling advancements, and policy interventions, aimed at reducing their ecological footprint without compromising patient safety.

## 2. Overview of Single-Use Plastics in Ophthalmology

### 2.1. Drivers and Constraints of Single-Use Eye Drop Packaging

Single-use plastic eye drop bottles became standard in ophthalmology due to infection control priorities and the practicality of disposables [3, 8–12]. Regulatory bodies continue to support these formats because they reduce the risk of microbial contamination, especially in high-risk settings [3, 8, 13–16]. Refilling or reusing containers is typically prohibited, as maintaining sterility cannot be guaranteed outside of controlled manufacturing environments. Even though multidose preservative-free bottles have been shown to be safe under certain conditions, adoption remains limited due to legal liability and strict compliance expectations [16, 17]. Additionally, the recycling of medical-grade plastics faces regulatory and material purity challenges, further limiting sustainable alternatives [3, 15]. As a result, infection control priorities and regulatory frameworks continue to reinforce single-use practices despite growing environmental concerns.

## 3. Environmental Impact and Waste Management Challenges

### 3.1. Waste Generation and Recycling Infrastructure

The small size and multimaterial design of eye drop bottles, which often combine low-density polyethylene (LDPE), high-density polyethylene (HDPE), adhesives, and labels, make them difficult to recycle [18]. Conventional recycling systems, particularly automated material recovery facilities (MRFs), are optimized for larger, uniform plastics and often miss or reject ophthalmic packaging altogether [16, 17, 19–21]. As a result, most eye drop bottles are ultimately disposed of through landfilling or incineration [16, 17]. For example, in the context of dry eye disease, where long-term use of lubricating eye drops generates substantial plastic waste, only 8.7% of this waste is actually recycled, while the vast majority is either incinerated or sent to landfills [22].

Recycling challenges are compounded in clinical settings by inadequate infrastructure, unclear protocols, and inconsistent staff engagement [15, 23]. These limitations reduce the likelihood that eye drop waste is properly recovered, highlighting a need for more targeted collection strategies and redesign of packaging to support recyclability.

### 3.2. Environmental Impact and Carbon Footprint of Eye Drop Bottles Across Conditions

Eye drop bottles contribute to greenhouse gas emissions throughout their life cycle, from raw material extraction and manufacturing to packaging, global distribution, and disposal [16, 24, 25]. While individually small, their cumulative environmental impact is substantial. For instance, in the context of dry eye disease, the annual carbon footprint of treatment has been estimated at 7 kg of CO_2_ per person [22]. Another study on dry eye treatments estimated that if 200 million people worldwide used single-dose vials of artificial tears four times daily, this practice alone would generate 22,600 tonnes of plastic waste per month, highlighting the significant carbon footprint of ophthalmic plastics [16, 17].

Similarly, cataract surgery, one of the most frequently performed procedures globally, accounts for approximately 10 million surgeries annually [26]. Postoperative care typically involves multiple eye drop medications, including antibiotics, steroids, and nonsteroidal anti-inflammatory drugs (NSAIDs). Even with a conservative estimate of one bottle per medication per patient, this translates to 30 million discarded plastic bottles annually from cataract surgery alone [27]. Given that a typical empty 10 mL eye drop bottle weighs approximately 6.5 g, this amounts to an estimated 195 metric tonnes of plastic waste each year, highlighting a significant environmental burden associated with postoperative care [17]. At the individual clinic level, an ophthalmologist managing 20–40 postoperative cataract patients per week would be responsible for generating approximately 3120–6240 discarded plastic bottles per year, contributing approximately 20–41 kg of plastic waste annually.

In the case of glaucoma, which affects around 80 million people worldwide, long-term management often relies on daily eye drop use to control intraocular pressure. If just 25% of glaucoma patients (20 million individuals) each use a 3 g 5 mL bottle containing 2.5 mL of solution per month for bilateral treatment, this amounts to 240 million plastic bottles used annually, generating 720 metric tonnes of plastic waste per year [17, 28–31]. At the clinic level, an ophthalmologist seeing 40–60 glaucoma patients per week would oversee the use of approximately 2080–3120 plastic eye drop bottles annually, equating to roughly 6–9 kg of plastic waste per year.

Beyond raw plastic production, energy-intensive sterilization and shipping practices also raise the overall environmental toll [25]. If waste management relies on incineration, further emissions, some potentially toxic, add to the problem. Ultimately, as global demand for ophthalmic treatments continues to rise, these figures underscore the pressing need for sustainable packaging solutions, improved drug delivery methods, and enhanced recycling initiatives to mitigate the environmental impact of single-use plastics in eye care.

## 4. Innovative Solutions and Future Directions

Addressing ophthalmic plastic waste requires a multifaceted approach that tackles product design, specialized recycling, and policy reforms.  Table 1 presents an overview of some of the approaches available.

### 4.1. Product Redesign and Material Innovation

Optimizing the design of eye drop bottles presents a direct opportunity to reduce negative environmental impact. One approach under investigation is the development of biodegradable and compostable materials that degrade more rapidly than conventional plastics [33]. However, most commercially available biodegradable plastics require specific industrial composting conditions, which are not widely accessible [34]. Additionally, the higher production costs associated with these materials pose a significant barrier to large-scale adoption, one that favors single-use plastics [34]. The term “biodegradable” can also be misleading, as some materials degrade only under controlled conditions and may persist in environments lacking the necessary composting infrastructure [44].

Multidose preservative-free systems offer another promising avenue. Single-dose vials use up to eight times more plastic, and each unit is discarded after a single use [16]. In contrast, properly designed multidose preservative-free eye drops have been noted to safely last for three to 6 months without contamination risk [17]. Such an approach may help prioritize patient safety, as many regulatory agencies dictate, while also significantly decreasing the number of plastic bottles produced, thereby also addressing cost-related factors. A recent multisociety position paper written by the Ophthalmic Instrument Cleaning and Sterilization Task Force has further endorsed the safe use of multidose topical medications across patients in surgical settings when proper guidelines are followed, supporting and highlighting the potential to reduce drug waste and carbon footprint in ophthalmic surgery [45].

Alternative drug delivery systems may further minimize reliance on bottle-based packaging. Innovations such as punctal plug-based drug delivery offer promising methods for administering ophthalmic medications while reducing plastic consumption [35]. Punctal plugs, which are small biocompatible devices inserted into the tear ducts, may be embedded with sustained-release medications, allowing for continuous drug delivery over weeks or months without the need for daily eye drops [35]. This technology not only improves patient adherence by eliminating the need for multiple daily applications but also significantly decreases plastic waste associated with conventional dropper bottles. However, before widespread implementation, these innovations must undergo rigorous evaluations for feasibility, cost-effectiveness, safety, and efficacy. If successfully integrated into clinical practice, these advancements could contribute to a substantial reduction in ophthalmic plastic waste while enhancing treatment outcomes.

### 4.2. Advanced Recycling Initiatives and Infrastructure

Since conventional recycling systems overlook or discard small plastic vials, specialized take-back programs have emerged as a critical step toward sustainability [16, 21]. Existing programs such as Bausch & Lomb's Biotrue Eye Care Recycling, which provide dedicated recycling streams for ophthalmic products, combined with institutional willingness presents a model that may be scaled effectively. Dedicated bins placed in clinics and pharmacies, paired with clear signage and regular staff training, can guide both patients and providers to deposit used items (e.g., single-dose vials and caps) in a single, well-marked location [37]. Once collected, these materials would be transferred to specialized facilities that can properly sort and recycle them, thereby bypassing the shortcomings of traditional municipal recycling centers [36]. Notably, these specific ophthalmology initiatives, like Bausch & Lomb's, have already diverted more than 94 million units of eye care waste, totaling over half a million pounds, from the waste stream in the United States alone [46]. Yet, expanding these programs may involve some hurdles. For instance, there are logistical and financial costs to consider: separate collection bins, patient education materials, and consistent shipping to specialized centers, especially with larger volumes that would exist with institutional collection.

Nevertheless, such an approach would not only prevent significant amounts of plastic from entering landfills and marine environments but also yield valuable data for future policy and product design. By tracking volumes, types of waste, and user participation, institutions can refine their recycling protocols and manufacturers can optimize packaging for easier recyclability. Additionally, in the case of chronic conditions such as glaucoma, where patient compliance with medication regimens is a well-documented challenge, monitoring the return of used eye drop bottles could serve as an indirect measure of adherence [47]. These data could help clinicians identify patients who may require additional support in managing their treatment. Moreover, the high visibility of these collection programs could foster a culture of sustainability among patients, clinicians, and staff. Ultimately, integrating specialized recycling programs into routine ophthalmic care could represent a practical method to advance environmental stewardship while maintaining patient safety, clinical efficiency, and improved treatment adherence.

### 4.3. Behavioral, Institutional, and Policy Interventions

Technological advances alone will not suffice; a systemic shift in behavior and policy is equally important. Educational campaigns targeting both healthcare professionals and patients can foster a culture of sustainability [38]. By integrating sustainability messages into patient discharge materials and clinical training programs, institutions can raise awareness about the environmental impacts of single-use devices and the importance of proper waste segregation.

An example of this type of institutional commitment is powerfully demonstrated in low- and middle-income countries, where specialized recycling infrastructure may be limited and sustainability models prioritize resource efficiency. The Aravind Eye Care System in India, for example, has developed a high-volume, low-cost model for cataract surgery that is also highly sustainable [32]. They achieve this by optimizing many steps of the clinical process. This optimization includes the safe reuse of surgical instruments, meticulous waste segregation, and the use of multidose eye drops, allowing Aravind to generate only a fraction of the waste and carbon emissions per procedure compared to hospitals in higher-income countries. This approach, driven by the necessity of providing affordable care at scale, demonstrates a powerful alternative to resource-intensive, disposable-heavy practices and serves as a model for reducing the environmental footprint of ophthalmology globally [32].

Building on the principles of such models, hospitals and clinics in any setting can drive change by adopting internal sustainability standards. These standards can prioritize the procurement of eco-friendly products and the implementation of robust recycling programs. However, while institutional actions are critical, systemic change is often accelerated by government policies and regulatory frameworks. Extended Producer Responsibility (EPR) policies—which hold manufacturers accountable for the end-of-life management of their products—are gaining traction globally, from the European Union and North America to Asia and countries in Africa [39–41, 48, 49]. These policies incentivize manufacturers to design products that are easier to recycle, thereby reducing the overall environmental impact.

This momentum is being coordinated at a global level by international collaborations and standard-setting organizations working to manage plastic pollution in healthcare [43]. In ophthalmology, EyeSustain is a leading global coalition dedicated to promoting environmentally sustainable practices [42]. By providing resources, education, and advocacy efforts, EyeSustain aims to help ophthalmic institutions reduce waste, implement greener practices, and adopt sustainable solutions without compromising patient care. As standards from these collaborations emerge, they may help harmonize efforts across different regions and facilitate the broader adoption of relevant recycling protocols. Through a combination of product innovation, improved infrastructure, behavioral change, and policy reform, ophthalmology can contribute to broader efforts to reduce plastic pollution in healthcare.

## 5. Discussion and Conclusion

### 5.1. Integrating Sustainability With Safety: Future Directions and Recommendations

Eye drop bottles reflect the broader challenge of balancing infection control with environmental sustainability [3]. While single-use packaging has historically minimized contamination risks, emerging evidence, as described in Section 4.1, confirms that sustainable alternatives can uphold these critical safety standards when properly implemented [17]. Similarly, specialized collection programs offer a practical way to recover ophthalmic plastic waste, converting it from an environmental burden into a recyclable resource [36, 37].

Further progress in reducing ophthalmic plastic waste hinges on systematic, evidence-based approaches. First, standardized laboratory testing of alternative materials, such as bioplastics or redesigned polymers, should be conducted under clinically relevant conditions to determine their safety, durability, and true biodegradability. Once promising materials are identified, smaller-scale clinical evaluations can then assess whether they maintain sterility and usability over time. Parallel life cycle assessments should compare these new packaging options with conventional single- and multidose bottles, quantifying both infection risk and carbon footprints to guide informed decision-making. Finally, pilot recycling programs in diverse settings (e.g., large hospital systems, smaller clinics, and community pharmacies) can generate practical insights on effective waste collection, sorting, and transport methods. This phased approach, starting with controlled materials testing and advancing through real-world clinical studies and targeted recycling initiatives, can clarify cost-effectiveness, ensure patient safety, and streamline adoption of more sustainable ophthalmic packaging.

Regulatory bodies, manufacturers, and healthcare systems must cooperate to examine infection risks and streamline approvals for lower-waste bottle designs and alternative drug delivery methods. Policymakers can facilitate these transitions by establishing standards for non-single-use plastics and implementing EPR frameworks that reward sustainable design choices. Hospitals and clinics can incorporate environmental targets into procurement guidelines and encourage patient participation through targeted education on responsible disposal.

### 5.2. Conclusion

Reducing the environmental impact of single-use eye drop bottles in ophthalmology requires a coordinated approach that integrates product design improvements, enhanced recycling infrastructure, and regulatory policies. While single-use devices have historically played a critical role in infection control and patient safety, increasing evidence highlights their significant environmental impact [3]. Strategies to mitigate plastic waste include redesigning eye drop bottles to improve recyclability, expanding the use of multidose systems where appropriate, and developing specialized recycling programs. Implementing these measures alongside behavioral and policy interventions can promote both environmental sustainability and patient safety. Rather than being conflicting priorities, these objectives can be aligned through collaborative efforts among clinicians, manufacturers, and policymakers. By adopting sustainable innovations, the ophthalmic field can contribute to reducing plastic pollution while maintaining high standards of care.

## Figures and Tables

**Table 1:** Design, recycling, and policy interventions to promote sustainable ophthalmic packaging.

Strategy area	Example actions	Key advantages	Key limitations/risks
Design	Multidose preservative-free bottles (including perioperative shared use under strict protocols) [16, 17, 32]	• Reduces the number of bottles used per patient and lowers overall drug waste	• Requires staff training and strict handling
		• Decreases the carbon footprint associated with packaging and disposal	• Demands adherence to stringent infection control protocols
		• Endorsed for safe use when strict guidelines are followed	• May raise medico-legal concerns in certain healthcare environments
	Biodegradable/compostable polymers for bottles/caps [33, 34]	• Potential for reduced environmental persistence compared to conventional plastics	• Most biodegradable plastics require specific industrial composting, which is not widely available
		• Can lower long-term waste burden if processed under appropriate industrial composting conditions	• Performance and cost trade-offs may limit scalability in ophthalmology
		• Aligns with broader trends toward eco-friendly packaging in healthcare	• Risk of greenwashing, where products are marketed as more environmentally sustainable than they truly are
	Alternative delivery (e.g., drug-eluting punctal plugs) [35]	• Reduces or eliminates the need for daily eye drop administration	• High up-front costs may limit accessibility
		• May enhance patient adherence, particularly for chronic conditions	• Requires careful device selection tailored to individual patients
		• Offers sustained drug release, potentially improving therapeutic outcomes	• Additional studies needed to confirm long-term safety, efficacy, and cost-effectiveness

Recycling/infrastructure	Clinic/pharmacy take-back streams (e.g., Biotrue with TerraCycle®) [36]	• Enables recovery of small-format items often missed by curbside recycling	• Requires dedicated logistics: setup of bins, shipping systems, and educational materials
		• Diverts ophthalmic plastics from landfills and incineration	• May be restricted to specific item types accepted by the program
		• Facilitates targeted recycling efforts tailored to eye care products	• Scalability depends on clinic participation/consistent user engagement
	On-site waste segregation combined with clear protocols and staff training [37, 38]	• Enhances the quality and accuracy of waste capture	• May interfere with clinical workflow and time efficiency
		• Establishes a solid foundation for effective specialized recycling	• Requires consistent staff engagement and reinforcement
		• Increases compliance with institutional and environmental standards	• Ongoing training and oversight needed to maintain effectiveness
	Design packaging for recyclability (e.g., use monomaterials, minimize labels and adhesives); adhere to minimum size thresholds for sorting [18]	• Simplifies material separation during recycling	• Very small items are often missed by automated sorting systems
		• Increases sorting efficiency and recovery rates at recycling facilities	• Packaging modifications may require coordination with manufacturers and suppliers
		• Enhances overall recyclability and yield from eye drop packaging	• May conflict with existing design, branding, or regulatory packaging constraints

Policy/behavior	Extended Producer responsibility (EPR) legislation for packaging [39–41]	• Shifts the responsibility and cost of end-of-life product management to manufacturers	• Implementation is often inconsistent across regions or countries
		• Encourages design of packaging that is easier to recycle	• Can impose significant administrative and compliance burdens on producers
		• Aligns incentives toward sustainable materials and reduced environmental impact	• Effectiveness depends on clear enforcement mechanisms and harmonized standards
	Professional coalitions and institutional standards (e.g., EyeSustain resources; greener procurement) [42]	• Provides field-specific guidance, education, and advocacy tailored to ophthalmology	• Participation is voluntary and uptake can be inconsistent
		• Encourages environmentally conscious practices such as sustainable procurement	• Success depends on leadership engagement and institutional commitment
		• Accelerates adoption of best practices through shared resources and peer alignment	• May require internal champions to drive sustained implementation
	Emerging global plastics treaties influencing medical plastic regulation [42, 43]	• May set unified upstream requirements for medical packaging design and sustainability	• Uncertainty remains regarding the scope, enforceability, and timelines
		• Encourages alignment between health, environmental, and manufacturing sectors	• Impact on healthcare-specific plastics will depend on how sectoral exemptions are addressed
